# Gold In-and-Out: A Toolkit for Analyzing Subcellular Distribution of Immunogold-Labeled Membrane Proteins in Freeze-Fracture Replica Images

**DOI:** 10.3389/fnana.2022.855218

**Published:** 2022-04-04

**Authors:** Debbie Guerrero-Given, Seth L. Goldin, Connon I. Thomas, Skylar A. Anthony, Diego Jerez, Naomi Kamasawa

**Affiliations:** The Imaging Center and Electron Microscopy Core Facility, Max Planck Institute for Neuroscience, Jupiter, FL, United States

**Keywords:** immunogold particles, image analysis, toolkit, freeze-fracture replica immunogold labeling, GUI (graphical user interface)

## Abstract

Integral membrane proteins such as ion channels, transporters, and receptors shape cell activity and mediate cell-to-cell communication in the brain. The distribution, quantity, and clustering arrangement of those proteins contribute to the physiological properties of the cell; therefore, precise quantification of their state can be used to gain insight into cellular function. Using a highly sensitive immunoelectron microscopy technique called sodium dodecyl sulfate-digested freeze-fracture replica immunogold labeling (SDS-FRL), multiple membrane proteins can be tagged with different sizes of immunogold particles at once and visualized two-dimensionally. For quantification, gold particles in the images must be annotated, and then different mathematical and statistical methods must be applied to characterize the distribution states of proteins of interest. To perform such analyses in a user-friendly manner, we developed a program with a simple graphical user interface called Gold In-and-Out (GIO), which integrates several classical and novel analysis methods for immunogold labeled replicas into one self-contained package. GIO takes an input of particle coordinates, then allows users to implement analysis methods such as nearest neighbor distance (NND) and particle clustering. The program not only performs the selected analysis but also automatically compares the results of the real distribution to a random distribution of the same number of particles on the membrane region of interest. In addition to classical approaches for analyzing protein distribution, GIO includes new tools to analyze the positional bias of a target protein relative to a morphological landmark such as dendritic spines, and can also be applied for synaptic protein analysis. Gold Rippler provides a normalized metric of particle density that is resistant to differences in labeling efficiency among samples, while Gold Star is useful for quantifying distances between a protein and landmark. This package aims to help standardize analysis methods for subcellular and synaptic protein localization with a user-friendly interface while increasing the efficiency of these time-consuming analyses.

## Introduction

Membrane proteins in the brain have unique roles in regulating the functions of neuronal and/or glial cells. Neuronal signaling is largely controlled by the movement of ions across the membrane, which is facilitated by ion channels embedded in the cell membrane. Neurotransmitter receptors located on cell membranes act to transmit chemical signals to downstream neurons in coordination with the activities of ion channels. Cell-type specific expression of these proteins, their subcellular location, clustering arrangement, and abundance are critical to determining the functional properties and diversities of cells (reviewed in Nusser, 2009, 2012), and research on these membrane proteins has expanded in recent decades. Methodologies ranging from molecular analysis to physiological measurements have been performed to understand the complex function of the membrane proteins in the brain (reviewed in Lujan and Ciruela, 2021).

Among the methodologies, sodium dodecyl sulfate-digested freeze-fracture replica immunogold labeling (SDS-FRL; Fujimoto, 1995) has the unique advantage of immunocytochemical identification of membrane proteins with immunogold particles on two-dimensionally visualized cellular membranes, and its high labeling efficiency allows us to perform quantitative analyses of the proteins by counting the number of gold particles. SDS-FRL has revealed membrane protein distributions both subcellularly and synaptically. For example, Na_*V*_1.6 has been shown to concentrate at the axon initial segment in CA1 pyramidal cells (Lorincz and Nusser, 2010); the voltage-gated calcium channel Ca_*V*_2.1 localizes in presynaptic specialization in small clusters (Nakamura et al., 2015; Lübbert et al., 2019; Rebola et al., 2019); Ca_*V*_2.1 also postsynaptically localizes in Purkinje cells showing a density-gradient from soma to distal dendrites (Indriati et al., 2013). Neurotransmitter receptors such as mGluR1α have been shown to perisynaptically colocalize with Ca_*V*_2.1 (Indriati et al., 2013); GABAB receptor colocalizes with GIRK2 and Ca_*V*_2.1 (Luján et al., 2018a); and mGluR1α colocalizes with SK2 and Ca_*V*_2.1 (Luján et al., 2018b). Furthermore, due to the high detectability of the epitopes in SDS-FRL, clustering phenomena of glutamate receptors have been reported in different cell types and brain regions (Tanaka et al., 2005; Masugi-Tokita et al., 2007; Antal et al., 2008).

To perform the quantitative and statistical analysis of labeled replicas, the accurate annotation of immunogold particles is imperative. Annotation had been done by hand until recently, which made it a meticulous and time-consuming task. In an effort to increase the efficiency of this process, several custom software packages were created to automate some aspects of the analysis, taking advantage of the rapid rise in computing power in the last decade. GoldExt performs quantitative analysis of the spatial distribution of receptor proteins in synaptic sites and features a graphical user interface with an integrated tool for multi-objective analysis of two-dimensional spatial patterns of the particles (Szoboszlay et al., 2017). However, demarcation of synapses and annotation of a single size of gold particle are entirely manual, and thus it is inefficient for large image analysis. Additionally, installation of GoldExt requires familiarity with the command prompt and specific versions of python and other package dependencies. GPDQ (Gold Particle Detection and Quantification) was designed to perform the automatic annotation and detection of immunogold particles in manually demarcated synaptic regions (Luján et al., 2018a). It also contains modules that compute the number of gold particles, determine nearest neighbor distance (NND) and clustering patterns, and simulate random particle distributions for comparative analysis of synaptic distributions of receptor proteins. The recently reported software Darea (Deep learning-assisted replica image analysis suite) expanded the functionality of GPDQ and improved its performance by integrating automated demarcation of pre- and post- synaptic areas and annotation of an unlimited number of particle sizes, allowing automated measurements of labeling density and particle distributions (Kleindienst et al., 2020). Nevertheless, like its predecessor, Darea is designed primarily to handle small synaptic images; large, irregularly shaped regions of interest (ROIs) are difficult or impossible to analyze. Moreover, basic familiarity with Python, Matlab, and CUDA is required to install Darea.

In an effort to expand automated analysis to be inclusive to subcellular protein distribution analysis, we recently developed a deep learning-based program called Gold Digger, which automatically annotates immunogold particles in large images (Jerez et al., 2021). Unlike GoldExt, it is capable of annotating up to 3 different sizes of immunogold particles and was designed to annotate very large ROIs, such as an entire cell soma or proximal dendrite, and outputs the XY coordinates of gold particles into a comma separated value (CSV) file format. However, it must be run within a Linux environment, which may be inconvenient for some users. Commercially and non-commercially available image analysis software such as Amira (Thermo Fisher Scientific, Waltham, MA, United States), Imaris (Oxford Instruments, Abingdon, United Kingdom), or Dragonfly [Object Research Systems (ORS) Inc., Montreal, QC, Canada] have also recently been equipped with machine learning modules which can be used for annotation of relatively large labeled replica images under a Windows environment. Regardless of the method used for annotating particles, a need now exists for user-friendly software that can perform particle distribution analysis without image size restrictions.

With the goal of having a standardized, comprehensive, and flexible analysis method, we present a new program called Gold In-and-Out (GIO). GIO can handle small or large image sizes with its user-friendly graphic interface, which makes it suitable for both synaptic and subcellular analysis. This package contains workflows for several basic immunogold particle analyses such as NND of the particles, clustering, and cluster separation. We also developed two workflows called Gold Rippler and Gold Star for analysis of particle distributions relative to a landmark feature, such as dendritic spines or other labeled proteins. For the purposes of statistical analysis, GIO automatically compares the real particle distribution to a simulated random distribution.

## Methods

### Animals

All animal procedures used in this study were approved by the Max Planck Florida Institute for Neuroscience Animal Care and Use Committee. Sample preparation was performed as previously described (Dong et al., 2018; Lübbert et al., 2019; Jerez et al., 2021).

In brief, for samples of cerebellum, two adult C57BL/6 mice (8 weeks old) were anesthetized with a cocktail of ketamine/xylazine (ketamine 100 mg/kg and xylazine 10 mg/kg, i.p.) and perfused transcardially with 0.9% NaCl followed by 4% paraformaldehyde in 0.1 M Sorensen’s phosphate buffer (PB, pH 7.4). For samples of brain stem, one C57BL/6 wild-type mouse at postnatal day 7, and two mice at postnatal day 7 expressing myristolated EGFP with overexpressed Ca_*V*_2.1 at the calyx of Held (Lübbert et al., 2019) were anesthetized with Tribromoethanol (250 mg/kg of body weight, i.p.) and perfused transcardially with phosphate-buffered saline followed by 4% paraformaldehyde and 0.2% picric acid in 0.1 M PB. The brain stem containing calyx of Held from the C57BL/6 wild-type mouse was used to generate the data for Ca_*V*_2.1 and Munc13-1 colocalization, and brain stems from animals overexpressing Ca_*V*_2.1 were used for other data sets.

### Replica Labeling and Imaging

The brain regions of interest were dissected after fixation and 130–150-μm-thick slices were sectioned using a vibratome (Leica VT1200, Leica, Wetzlar, Germany). Slices were serially incubated in 10, 20, and 30% glycerol at 4°C for cryoprotection. The tissue was trimmed to contain the region of interest and frozen using a HPM100 high-pressure freezing machine (Leica, Wetzlar, Germany). The frozen samples were fractured into halves using a double replication device at –140°C. They were replicated with layers of 2–3 nm carbon followed by 2 nm carbon-platinum and then 30–40 nm carbon using a JFDV or JFDII freeze-fracture machine (JEOL/RMC Boeckeler, Tucson, Arizona). The tissue was dissolved in a digesting solution containing 2.5% sodium dodecyl sulfate (SDS), 20% sucrose, and 15 mM Tris-HCl (pH 8.3) with gentle agitation (82.5°C for 19 h, or for nine or 18 h for the digestion comparison experiment). After digestion, the replicas were washed and blocked with 4% bovine serum albumin and 1% cold fish skin gelatin in 50 mM Tris-buffered saline. The replicas were then incubated at room temperature for overnight with following antibodies per each experiment: (1) cerebellum replicas—a guinea pig anti Ca_*V*_2.1+ channel P/Q-type alpha-1A antibody (Synaptic Systems, Göttingen, Germany; 152–205 at 0.7 μg/ml), (2) brain stem replicas used for the large terminal image analysis—a guinea pig anti Ca_*V*_2.1+ channel P/Q-type alpha-1A antibody (Synaptic Systems, Göttingen, Germany; 152–205 at 0.7 μg/ml) and rabbit anti GFP antibody (Abcam, Cambridge, United Kingdom; ab6556 at 1μg/ml), 3) brain stem replicas used for Ca_*V*_2.1 and Munc13-1 localization analysis—the same guinea pig anti Ca_*V*_2.1+ channel P/Q-type alpha-1A antibody and a rabbit anti Munc13-1 antibody (Synaptic Systems, Göttingen, Germany; 126–103 at 1 μg/ml). After washing, the replicas were incubated at room temperature for 18 h with a combination of the following secondary antibodies at 1:30 dilution: 1) cerebellum replica—a donkey anti-guinea pig IgG conjugated to 12 nm gold particles (Jackson ImmunoResearch, West Grove, Pennsylvania; 706-205-148), 2–3) brain stem replicas—a donkey anti-guinea pig IgG conjugated to 12 nm gold particles (Jackson ImmunoResearch, West Grove, Pennsylvania; 706-205-148) plus a donkey anti-rabbit IgG conjugated to 6 nm gold particles (Jackson ImmunoResearch, West Grove, Pennsylvania; 711-195-152). The replicas were washed and placed on copper aperture grids and examined with a Tecnai G2 Spirit BioTwin transmission electron microscope (Thermo Fisher Scientific, Waltham, MA, United States) at 100 kV acceleration voltage. Images were taken with a Veleta CCD camera (Olympus, Tokyo, Japan) controlled by a TIA software (Thermo Fisher Scientific, Waltham, MA, United States), at a 43,000 × magnification. Images were tiled and minimum adjustments of brightness and contrast were done in Photoshop (Adobe CS6, San Jose, CA, United States). Tiles were merged and saved as tiff files for analysis.

### Deep Learning-Based Annotation of Gold Particles and Regions of Interest From Sodium Dodecyl Sulfate-Digested Freeze Fracture Replica Immunogold Labeling Images

Annotation of gold particles was done one of two ways, both using deep learning approaches. A network we developed previously, Gold Digger (Jerez et al., 2021), was used to automatically annotate all gold particles on the selected membrane region of interest. Immunogold-labeled replica images along with a corresponding “mask” for the area of interest were uploaded to the software. For this approach, the mask was created manually in Photoshop (Adobe CS6, San Jose, CA, United States). Each of the gold particles detected was given an identification number, and XY coordinates were exported as a CSV file. With Gold Digger, users have the ability to open the image with the corresponding CSV output in FIJI (Schindelin et al., 2012) to manually correct annotation errors by deleting false positives and adding false negatives.

Dragonfly version 2021.3 for Windows [Object Research Systems (ORS) Inc., Montreal, QC, Canada; software available at http://theobjects.com/dragonfly] was also used to automate gold particle annotation. Like Gold Digger, both the replica and mask images were loaded into the software. A U-Net deep learning architecture was trained with several manually annotated replica images split into patches of 32 × 32 pixels to segment two different sizes of immunogold particles. For network training, ∼7 μm^2^ of Purkinje soma membrane was used in total, and this network was also used to detect immunogold particles on calyx membranes. A second network was trained in Dragonfly to create an ROI mask for the Purkinje cell protoplasmic face (P-face). A LinkNet architecture was trained using manually segmented P-face membrane images split into patches of 512 × 512 pixels. Ten cellular profiles of various sizes (three soma profiles and seven dendrites) were used to train the Purkinje cell network, totaling ∼300 μm^2^ of membrane. This training set was supplemented with a small dataset of calyx of Held membrane for specific segmentation of calyx profiles. For both networks, a subset of the training data (20%), selected randomly, was set aside for use as a validation dataset. The networks were set to be trained for 100 epochs and ended when early stopping conditions were met. The network could successfully identify the P-face of Purkinje cell dendrites while excluding small patches of exoplasmic face (E-face) membranes. Any segmentation errors for gold particles or P-face membranes were manually adjusted using the Dragonfly segmentation interface.

### Development of User-Friendly Software for Immunogold Particle Analysis

We developed GIO with the goal of incorporating multiple tools for immunogold particle analysis in freeze-fracture replica images into a single software package. GIO was written in Python 3.8 using the graphical user interface library PyQT5. Multi-threading is utilized to minimize runtime. The package is compiled to a single executable file (<700 MB), making it easy to install on Windows computers. Each analysis tool includes adjustable parameters that allow further customization when needed, which can be modified in the graphical user interface without any need for coding knowledge. GIO also utilizes Seaborn, a Python visualization package, to allow users to adjust the color scheme of generated outputs to fit their preference. GIO is compatible with both Windows and macOS. In our figures, some colors were enhanced slightly in Photoshop to improve visibility.

Users begin by uploading (1) a gold-labeled replica image in TIFF format, (2) a mask demarcating the ROI, also in TIFF format, (3) a CSV file containing the X coordinates of annotated gold particles in a column titled “X” and the Y coordinates in a column titled “Y,” and optionally, (4) a CSV file containing the XY coordinates for landmark features, also in columns titled “X” and “Y,” respectively. These files can be uploaded individually, or alternatively, GIO offers the option to upload these materials simultaneously using the “upload folder” option. A folder containing all of the above can be selected, and GIO will automatically populate each field with the correct file. This option requires that filenames include the following keywords for accurate identification: “image” for the labeled replica, “mask” for its corresponding mask, “gold” for the CSV file containing annotated particle coordinates, and “landmark” for the CSV file containing landmark coordinates. Once the appropriate input files are uploaded using either method, the user proceeds by assigning their desired output destination folder, and selecting their desired workflows. Finally, because the program performs all internal calculations using pixel units, the unit conversion parameter must be set accordingly to generate results in the desired units. When the analysis is complete, a CSV file will be generated for each selected workflow containing numerical results. Simultaneously, GIO presents a graph for each workflow along with a graphical representation of particles in the result tab. GIO is able to generate a pseudo-random population of gold particles and distribute them in the area of interest. The number of generated particles is matched to the number of real particles by default, but can be modified in the menu page. When the “display random coords” box is checked, the calculated results of the original biological distributions are presented alongside the randomized reference population. Individual workflows are designed as described in the following paragraphs.

#### Generation of Random Particle Distribution

A pseudo-random distribution of immunogold particles is automatically generated for reference purposes using Python random randint, which creates a random float in the range (0.0, 1.0) and is itself based on the widely used deterministic “Mersenne Twister.” The program accepts the boundaries of a selected image mask and creates pseudo-random integer coordinates within the range, keeping the particles within the specified area where coordinates should be found. A 5-pixel minimum distance to other gold particles is applied when the random distribution is generated to reduce occurrences of overlap which could not occur in a real sample. The implementation generates either a set of N coordinates or an equivalent count to the number of gold particles in the CSV file by default. This value can be modified on the main page for specific purpose comparisons.

#### Nearest Neighbor Distance of Gold Particles

The NND for all gold particles is calculated in GIO. The location of each gold particle in the replica image is represented by an XY coordinate pair in the user-uploaded CSV file; with these coordinates, GIO can determine NND using the Euclidean distance formula, where the distance (*d*) between two particles (*p*1 and *p*2) is given by


$$d\,\left(p\,1,p\,2\right)=\sqrt{{\left(X\,2-X\,1\right)}^{2}+{\left(Y\,2-Y\,1\right)}^{2}}.$$

This formula is iterated for each particle in comparison to all other particles, and the NND is then identified as the minimum calculated distance between a given particle and its nearest neighbor. Results are then compiled into a CSV file, which contains the coordinates of each gold particle, the coordinates of its nearest neighbor, and the NND. Additionally, GIO outputs a second CSV file for a randomly generated control distribution, a histogram of NND results, and a visual representation of those results, where NNDs are illustrated by gradient-color-coded lines between particles and overlaid onto the original image. Users can customize which data are included in the histogram and overlay by selecting “show real distribution,” “show random distribution,” or both. If needed, the histogram bin size can also be adjusted.

#### Cluster Analysis of Gold Particles

Cluster analysis of gold particles is performed with the hierarchical method (Gower and Ross, 1969). GIO finds clusters of gold particles and sorts those into groups using sklearn AgglomerativeClustering, a form of agglomerative clustering within the scikit-learn python package (Pedregosa et al., 2011). Euclidean distance was used to calculate the affinity metric. GIO mathematically calculates clusters using a given distance threshold. By default, and for most cases, the distance threshold method is used for replica image analysis. GIO creates a circle of set radius length centered on each gold particle, then sorts particles into groups (i.e., clusters) based on whether their circles overlap. In our analyses, we set the clustering radius to 30 nm to account for labeling uncertainty as in our previous analysis of Ca_*V*_2.1 localization (Lübbert et al., 2019). Clusters are then assigned identification numbers and the area of each cluster is calculated as the union of all overlapping circles when this option is selected. The results are summarized in two CSV files. One contains the cluster ID, number of gold particles in the cluster (cluster size), and the cluster area; the other provides the coordinates of each particle along with the ID of the cluster it belongs to. The colored boundaries of the clusters can be displayed on the replica image by selecting “draw clust area.”

#### Separation Between Clusters

GIO is able to measure the separation of clusters—i.e., the distance from an individual cluster to its nearest cluster (Althof et al., 2015). It determines the centroid of each cluster by finding the average of both X and Y values of all points in the cluster. For this analysis, clusters are defined as containing two or more gold particles. This criterion can be modified in the menu page depending on the user’s purpose. GIO then performs the same NND calculation as described in section “Nearest Neighbor Distance of Gold Particles” on all clusters that meet the set criteria. The results are summarized in two CSV files. One contains the coordinates of each particle along with the ID of the cluster it belongs to; the other provides the centroid of each cluster, its nearest cluster, the distance between them, and the cluster ID.

#### Gold Rippler

Gold Rippler is a unique workflow developed to analyze the positional bias of the gold particles. GIO takes an input of particle coordinates uploaded in the first CSV file, and the coordinates of “landmarks” (e.g., center of dendritic spines) uploaded in a second CSV file, which were annotated manually in FIJI or Dragonfly. It then extends a series of circles, termed “ripples,” from the centroid of each landmark. These ripples increase in radius by a user-defined step size (60px by default) from an initial radius (50px by default), for a set number of steps (10 by default). Each subsequent ripple includes the area of the previous one. It then calculates the “Landmark Correlated Particle Index (LCPI)” for each bin, given by the equation


$$L\,C\,P\,I=\frac{\mathrm{proportion}\,\mathrm{of}\,\mathrm{particles}\,\mathrm{covered}\,\mathrm{by}\,\mathrm{the}\,\mathrm{ripple}}{\mathrm{proportion}\,\mathrm{of}\,\mathrm{ROI}\,\mathrm{covered}\,\mathrm{by}\,\mathrm{the}\,\mathrm{ripple}}.$$

The result shows whether the distribution of gold particles tends to be uniformly spread, closer to, or farther from the landmarks. When gold particles are evenly distributed, the proportion of gold particles encompassed by each ripple should be equivalent to the proportion of membrane area encompassed by each ripple, yielding an LCPI of 1. The results are summarized in a CSV file containing the ripple radius, percentage of the total number of particles contained within, percent of the total ROI area contained within, LCPI values, and total number of particles in the entire ROI.

#### Gold Star

Gold Star calculates the NND between immunogold particles and landmarks by finding the smallest Euclidean distance between each particle and its closest landmark using the primary and secondary CSV files loaded. It is similar to Gold Rippler, but it provides the distribution of distances from gold particles to the nearest landmark. The results are summarized in a CSV file containing the coordinates of the particles, coordinates of the nearest landmark feature, and the distance between the two.

### Statistical Analysis

Statistical comparisons were made using R version 4.1.1 as well as the R companion package for calculating effect sizes (R Core Team, 2020; Mangiafico, 2021). Graphs of compiled data from several ROIs were created using Prism version 9.0.0 (GraphPad software, San Diego, California). Wilcoxon signed-rank tests were used to compare real and random distributions for mean NND, gold particles per cluster, particle density, cluster area, and cluster separation of overexpressed Ca_*V*_2.1 channels on calyx of Held terminals (*n* = 6). This was also performed for the same analysis of Ca_*V*_2.1 channels on Purkinje cell proximal dendrites (*n* = 11), as well as for landmark NNDs calculated using Gold Star. All random distributions contained the same number of particles within the ROI as the real distribution of the same ROI. An α of 0.05 was used for all statistical comparisons. Effect size is reported as the rank biserial correlation coefficient *r*, calculated using the wilcoxonPairedRC function, where values closer to –1 or 1 show a larger effect size (Mangiafico, 2021). For our analysis comparing Munc13-1 and Ca_*V*_2.1 particle density of the 9- and 18-h digestion conditions, we performed two-sample *t*-tests with Welch’s correction (n = 6). For analysis comparing Munc13-1 and Ca_*V*_2.1 with Gold Rippler, multiple comparisons *t*-tests, corrected with the two-stage step-up method with the desired False Discovery Rate set to 5% (Benjamini et al., 2006), were run on each bin to compare the mean LCPI of the 9- and 18-h digestion conditions. For comparison of mean particle-particle NNDs between the digestion conditions, a Mann-Whitney test was performed and the effect size is reported as the Glass rank biserial correlation coefficient *g*, calculated using the wilcoxonRG function, where values closer to -1 or 1 show a larger effect size. All means are reported ± the standard error (S.E.M).

## Results

With a simple user interface, GIO is able to accelerate time-consuming data analysis and helps standardize the analysis method. We tested GIO to analyze the subcellular distributions of overexpressed Ca_*V*_2.1 channels on calyx of Held terminals and compared the output with our previously published data (Lübbert et al., 2019), with which we manually analyzed the particle distributions of the same images using Fiji. We repeated this analysis for images of Purkinje cell dendrites labeled for Ca_*V*_2.1 channels. Additionally, we applied GIO for synaptic analysis of the colocalization of two presynaptic active zone proteins—Ca_*V*_2.1 and Munc13-1.

### Gold In-and-Out Functionality and Its Performance

**FIGURE 1:**
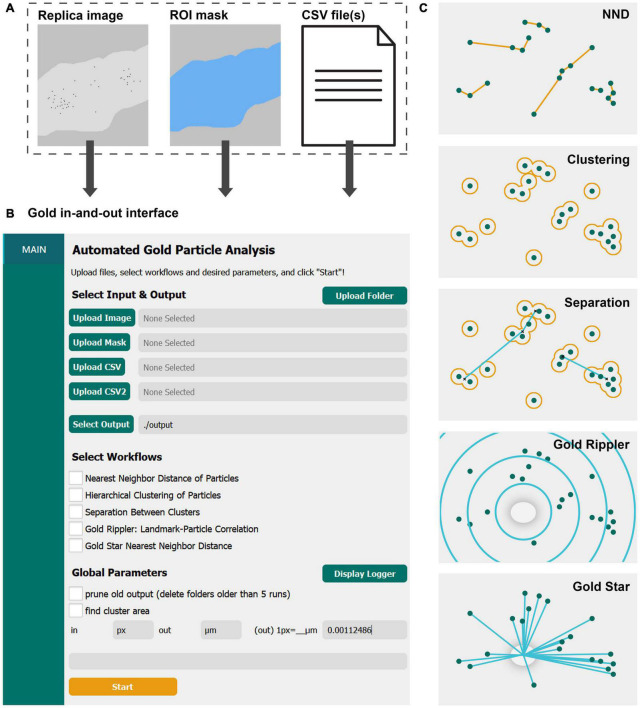
GIO is a user-friendly graphical interface that provides several workflows for quantitative spatial analysis of immunogold particles on freeze-fracture replica images. **(A)** GIO takes three inputs—a montaged image of an immunogold labeled replica, an ROI mask of the membrane of interest, and CSV files of the XY coordinates of up to two populations of gold particles or structural landmarks. **(B)** The main interface of GIO, showing the folder upload button, individual file upload fields, the output folder field, workflow selections, and global parameters. **(C)** An illustration of the different workflows available in GIO. NND allows for measurement of nearest particle distances. Clustering groups particles based on their proximity to one another using a circle of a set radius. Separation measures the NND of cluster centroids (clusters shown as having two or more particles). Gold Rippler bins particles within concentric circles, or ripples, based on proximity to a landmark feature to measure particle bias toward or away from it. Gold Star measures nearest distances of particles to a landmark feature. The cross-fracture of a dendritic spine (gray oval) is shown as the landmark feature for both Gold Rippler and Gold Star.

We designed GIO to include methods consistently applied to analyze membrane protein distributions in labeled replicas and create workflows for measuring NND of gold particles, clustering of gold particles, and separation between clusters of gold particles. In addition, we developed two new analysis workflows. Gold Rippler is used to analyze the bias of labeled proteins relative to biological landmarks such as dendritic spines, and Gold Star is used to measure the distances from individual particles to the closest landmark (Figure 1). For all workflows, the actual biological distribution of particles can be simultaneously compared to a mathematically generated random distribution.

To start image analysis, three files are loaded into GIO: A labeled replica image in tiff format, an ROI mask in tiff format representing the region of interest to analyze, and a CSV file of the particle coordinates that were obtained prior to running GIO (Figure 1A). When applying Gold Rippler or Gold Star, a second CSV file is uploaded containing the coordinates of landmark features (“CSV2” in Figure 1B). Using the main interface of GIO, users can set the output folder location, then select the workflows to be performed (Figure 1B). Global parameters such as output pruning, display of the logger, and cluster area calculations can be set as well. The user then sets an input and output scale based on the units and scale of the input images and particle coordinates. After clicking “Start,” the selected workflows are run and can be completed in less than a minute. When the optional function “find cluster area” was selected, the analysis required a few additional minutes depending on the number of particles in the image. The progress can be visually monitored with the optional function to “display logger,” which shows the line-by-line progress of the program. When it finishes, the result tabs of “NND,” “CLUST,” “SEPARATION,” “RIPPLER” and “GOLDSTAR” are shown below the “Main” tab in the left column, depending on which workflows are chosen. A visual representation of each workflow is shown in Figure 1C, and individual workflows are described in the following sections. For demonstration of its practical applications and to show anticipated results of the new program, we applied GIO to two different types of replica images; one from calyx of Held terminals in the brain stem for gold particles localizing on large flat membranes, and another for particles localizing on Purkinje cell dendrites with spines, representing curved and contoured membranes.

### Nearest Neighbor Distance of Gold Particles

**FIGURE 2:**
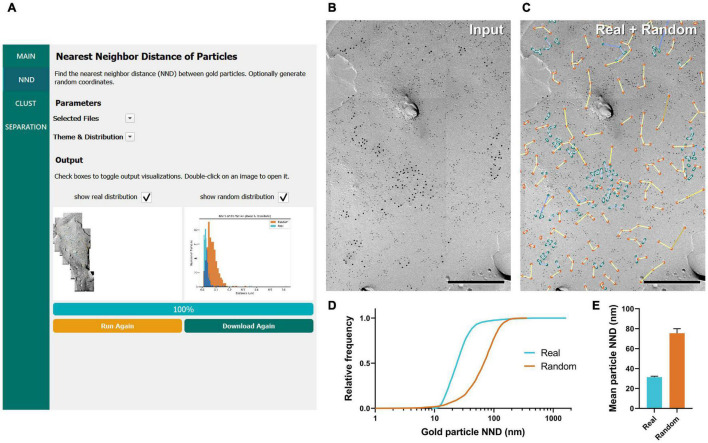
GIO workflow result of gold particle NND analysis of Ca_*V*_2.1 at the calyx of Held. **(A)** GIO interface showing the NND analysis workflow. Parameters such as the selected files and color theme can be changed here. The output for an ROI is displayed at the bottom of the interface; A labeled image of the ROI is displayed as well as a histogram of the particle NNDs for real and/or random distributions. Note that this is for one ROI only. **(B)** A portion of a calyx of Held ROI input image, with large 12 nm gold particles labeling Ca_*V*_2.1. Small 6 nm gold particles were used as a cell-specific marker. **(C)** The GIO output of the same image in **(B)**, with real particles labeled in green and randomly distributed particles in orange (superimposed). Lines representing particle NND pairs are also drawn on the output. **(D)** Cumulative frequency distribution of real and random particle NNDs pooled across the six ROIs. **(E)** Grand mean particle NND comparing real and random particle distributions. Error bars represent S.E.M. *n* = 6 ROIs. Scale bars: 500 nm.

Frequently, NND between gold particles on the replica image is the first parameter used to analyze the distribution of labeled proteins (Szoboszlay et al., 2017; Lübbert et al., 2019; Rebola et al., 2019; Karlocai et al., 2021). We implemented a basic particle NND analysis method that calculates the distance for all annotated gold particles to its nearest neighbor using the XY coordinates from the uploaded CSV file (see details in “Methods” section). The result is summarized into a new CSV file and exported to a designated folder. A color overlay image with lines between the closest particles, and circles at the centroid of each particle (Figure 2A bottom left, enlarged view in Figure 2C), as well as a histogram of particle NNDs (Figure 2A bottom right) are also exported. The NNDs of real particles are displayed as blue-colored lines by default (Figures 2B,C). A pseudo-random distribution of the same number of gold particles can be simultaneously created when the option is selected, presented with a separate color palette (yellow by default; Figure 2C). Users can modify the color palette for real and random distributions from the “Parameters” pull-down selection in the interface.

To demonstrate the efficacy of GIO, we analyzed six images from a dataset that we previously used to report the clustering arrangement of a voltage-gated calcium channel, Ca_*V*_2.1, in calyx of Held terminals to examine changes in distribution when the channel protein was overexpressed (Lübbert et al., 2019). To obtain the NND for all the annotated gold particles, GIO required less than 1 min per image, and allowed us to quickly compare real to random NND particle distributions, compared to ca. 2 min required for manual analysis using our in-house Excel macro. In general, particle NNDs spanned a wide range of values (real: 9 –1,649 nm, median = 24 nm, random: 1–361 nm, median = 68 nm; Figure 2D). The real distribution of Ca_*V*_2.1 channels had a significantly shorter mean particle NND compared to random (real mean: 31.41 ± 0.84 nm, random mean: 75.44 ± 4.57 nm; *W* = 21, *p* = 0.03, *r* = 1, *n* = 6 ROIs; Figure 2E). These data support previous observations that Ca_*V*_2.1 channels distribute non-randomly on the membrane (Nakamura et al., 2015; Lübbert et al., 2019).

### Clustering of Gold Particles

**FIGURE 3:**
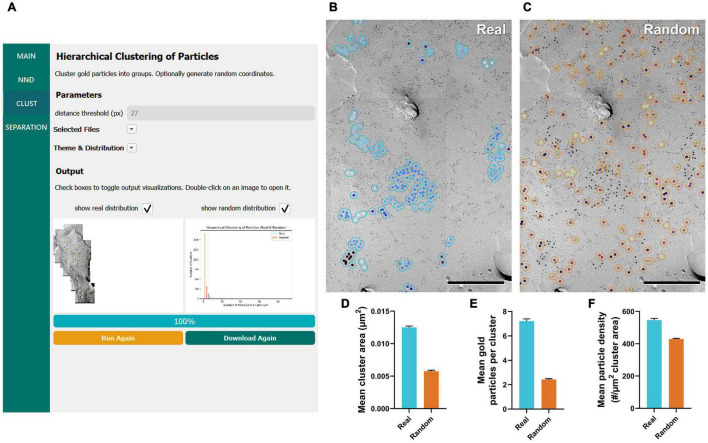
GIO workflow result of gold particle hierarchical clustering analysis of Ca_*V*_2.1 at the calyx of Held. **(A)** GIO interface showing the cluster analysis workflow. Parameters show additional fields for setting a distance threshold (in pixels). A labeled image of the ROI is displayed along with a histogram of the number of particles per cluster for real and/or random distributions. **(B)** The cluster analysis output for real particles, showing the same portion of ROI as in Figure 2B. **(C)** The cluster analysis output for randomly distributed particles superimposed on the same portion of ROI. **(D)** Grand mean area of clusters. **(E)** Grand mean number of gold particles per cluster. **(F)** Grand mean particle density within clusters. Error bars represent S.E.M. *n* = 6 ROIs. Scale bars: 500 nm.

The clustering of membrane proteins is another parameter that is of interest for understanding protein organization (Nakamura et al., 2015; Dong et al., 2018; Lübbert et al., 2019; Radulovic et al., 2020). By running the workflow of “Hierarchical Clustering of Particles,” gold particles are clustered into groups if they are within a certain distance of another particle (30 nm, or 27 pixels as the default; Figure 3A). Identification numbers of each cluster, including ones having a single particle, are exported into a CSV file. In addition, when “find cluster area” is selected in the main menu, the area of each cluster is also calculated. The outlined circles of each cluster are drawn and presented on the replica image together with or separately from the randomly generated clusters (Figures 3B,C).

Similar to our NND analysis, we compared the clustering arrangement of Ca_*V*_2.1 channels on calyx of Held terminals to that of a random distribution of particles with a set radius of 30 nm. We found that mean cluster area of real particles was consistently and significantly higher than that of a random distribution (real mean: 0.0125 ± 0.0002 μm^2^, random mean: 0.0058 ± 0.0004 μm^2^; *W* = 21, *p* = 0.03, *r* = 1, *n* = 6; Figure 3D). Additionally, the mean number of gold particles per cluster in the real distribution was consistently and significantly higher than random (real mean: 7.22 ± 0.18 particles, random mean: 2.43 ± 0.06 particles; *W* = 21, *p* = 0.03, *r* = 1, *n* = 6; Figure 3E). Mean particle density within a cluster followed the same trend (real mean: 546.4 ± 10.59 particles/μm^2^, random mean: 429.1 ± 3.90 particles/μm^2^; *W* = 21, *p* = 0.03, *r* = 1, *n* = 6; Figure 3F). These data support our NND analysis result that Ca_*V*_2.1 is clustering and provides an estimate of how many channel proteins exist in a given cluster.

In our previous work, we manually performed a similar analysis of clustering of Ca_*V*_2.1 proteins at calyx of Held terminals using in-house developed macros for Fiji and Excel (Lübbert et al., 2019); therefore, we took this opportunity to test the accuracy of GIO compared to manual analysis. We observed that the mean cluster area was consistently lower when calculated by GIO compared to manual, but this difference is negligible (GIO mean: 0.0125 ± 0.0002 μm^2^, manual mean: 0.0128 ± 0.0002 μm^2^; *W* = 21, *p* = 0.03, *r* = 1, *n* = 6; Figure 3D). Similarly, we saw a negligible yet consistently lower mean number of gold particles per cluster with GIO compared to manual (GIO mean: 7.22 ± 0.18 particles, manual mean: 7.39 ± 0.19 particles; *W* = 21, *p* = 0.03, *r* = 1, *n* = 6; Figure 3E). We saw no difference in the mean particle density within a cluster (GIO mean: 546.4 ± 10.59 particles/μm^2^, manual mean: 545.3 ± 10.94 particles/μm^2^; *W* = –11, *p* = 0.313, *r* = –0.524, *n* = 6; Figure 3F). We confirmed that GIO was considerably faster than manual methods for analysis; the clustering data sheet could be generated in 5 min for a large ROI (e.g., 1,244 gold particles, 215 clusters, in ca. 18 μm^2^, 400 Mb image), whereas manual analysis of the same ROI using our in-house macro in Fiji required about 2 h, because all gold particles needed to be selected by hand as part of the macro’s workflow.

### Separation Between Clusters

**FIGURE 4:**
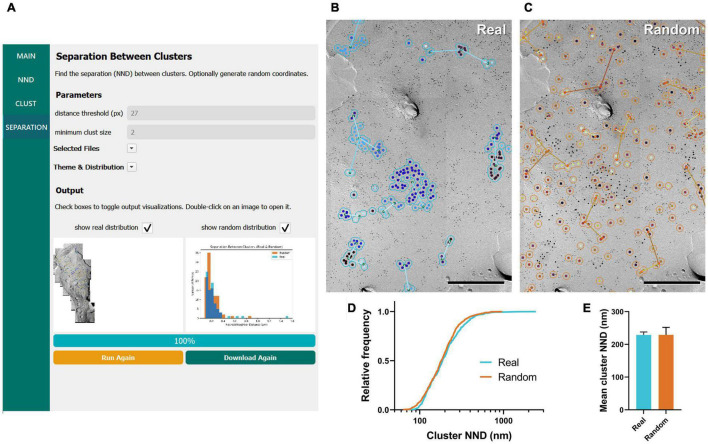
GIO workflow result of gold particle cluster separation analysis of Ca_*V*_2.1 at the calyx of Held. **(A)** GIO interface showing the cluster separation analysis workflow. Parameters show an additional field for setting the minimum cluster size, i.e., the minimum number of particles in a cluster. A labeled image of the ROI is displayed along with a histogram of the cluster nearest neighbor distances for real and/or random distributions. **(B)** The separation analysis output for real particles, showing the same portion of ROI as in Figure 2B. Lines are drawn connecting nearest clusters. **(C)** The separation analysis output for randomly distributed particles superimposed on the same portion of ROI. **(D)** Cumulative frequency distribution of real and random cluster NNDs pooled across the six ROIs. **(E)** Grand mean cluster NND. Error bars represent S.E.M. *n* = 6 ROIs. Scale bars: 500 nm.

Separation, defined as the NND between clusters, is another metric of interest when assessing the aggregation of clusters of membrane proteins (Althof et al., 2015). In the “Separation between clusters” workflow, GIO selects clusters containing two or more gold particles by default and calculates the NNDs between them using their centroid coordinates. Similar to the clustering workflow, several parameters can be set, such as the distance threshold and number of clusters to generate (Figure 4A). The separation of clusters is displayed in the output image, represented by colored lines, and a histogram is created to compare real and random distributions (Figures 4A–C). All numerical outputs are summarized in a CSV file and saved in the designated folder for further statistical analysis.

We applied the workflow of separation between clusters for the same calyx of Held replica images above, with a minimum cluster size of two particles. We observed no difference in the mean cluster NND between real and random (real mean: 228.87 ± 8.96 nm, random mean: 229.16 ± 22.66 nm; *W* = –7, *p* = 0.563, *r* = –0.333, *n* = 6; Figures 4D,E). This suggests clusters are randomly distributed on the membrane.

### Performance of Gold In-and-Out on Dendrites

**FIGURE 5:**
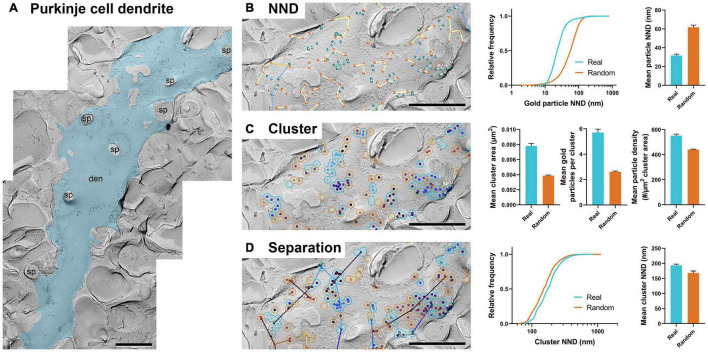
GIO can be applied to replica images of dendrites. **(A)** An image of a portion of Purkinje cell dendrite with 12 nm gold particles labeling Cav2.1. The dendrite ROI is shown as a blue overlay. sp = spine, den = dendrite. **(B)** GIO output for particle NND analysis of real and random distributions including a comparison of cumulative frequencies and mean particle NNDs. **(C)** GIO output for cluster analysis of real and random particle distributions, including grand mean cluster area, number of gold particles per cluster, and gold particle density within clusters. **(D)** GIO output for separation analysis of clusters of real and random particle distributions, including a comparison of cumulative frequencies and grand mean cluster NND. Error bars represent S.E.M. *n* = 11 ROIs. Scale bars: 500 nm.

We confirmed GIO’s usefulness on the calyx of Held terminal, which exhibits uncharacteristically large and flat membrane area in replicas. This is in contrast with other common neuronal features, such as dendrites, which are relatively thin, exhibit considerable curvature, and have a number of dendritic spines along their shaft. We therefore deemed it necessary to verify the effectiveness of GIO on dendrite profiles. For this, we used images of Purkinje cell dendrites labeled with immunogold for Ca_*V*_2.1 channels (Figure 5A).

We ran 11 images of dendrite segments and analyzed particle NND, clustering, and separation of clusters (Figures 5B–D) and confirmed the workflows provided meaningful numerical data. We compared the real and random distribution in each workflow. Mean particle NNDs differed significantly from random (real mean: 31.69 ± 1.48 nm, random mean: 61.79 ± 2.15 nm; *W* = 66, *p* = 0.001, *r* = 1, *n* = 11; Figure 5B). Mean cluster area, particles per cluster, and particle density were all significantly different from random (real mean cluster area: 0.0078 ± 0.0003 μm^2^, random: 0.0039 ± 0.0002; real mean particles per cluster: 5.7 ± 0.3 particles, random: 2.6 ± 0.05 particles; real particle density: 551.76 ± 11.79 particles/μm^2^, random: 440.74 ± 2.92 particles/μm^2^; all comparisons: *W* = –66, *p* = 0.001, *r* = –1, *n* = 11; Figure 5C). Separation of clusters was also significantly different between real and random, unlike what was seen in the calyx of Held (real mean: 193.48 ± 4.20 nm, random mean: 168.18 ± 6.58 nm; *W* = –60, *p* = 0.005, *r* = –0.909, *n* = 11; Figure 5D). These data suggest Ca_*V*_2.1 channels on Purkinje dendrites exhibit clustering as reported previously (Indriati et al., 2013). Furthermore, the clusters themselves showed inhomogeneous distribution, implying an unknown but particular biological association to a structural or functional factor.

### Gold Rippler

**FIGURE 6:**
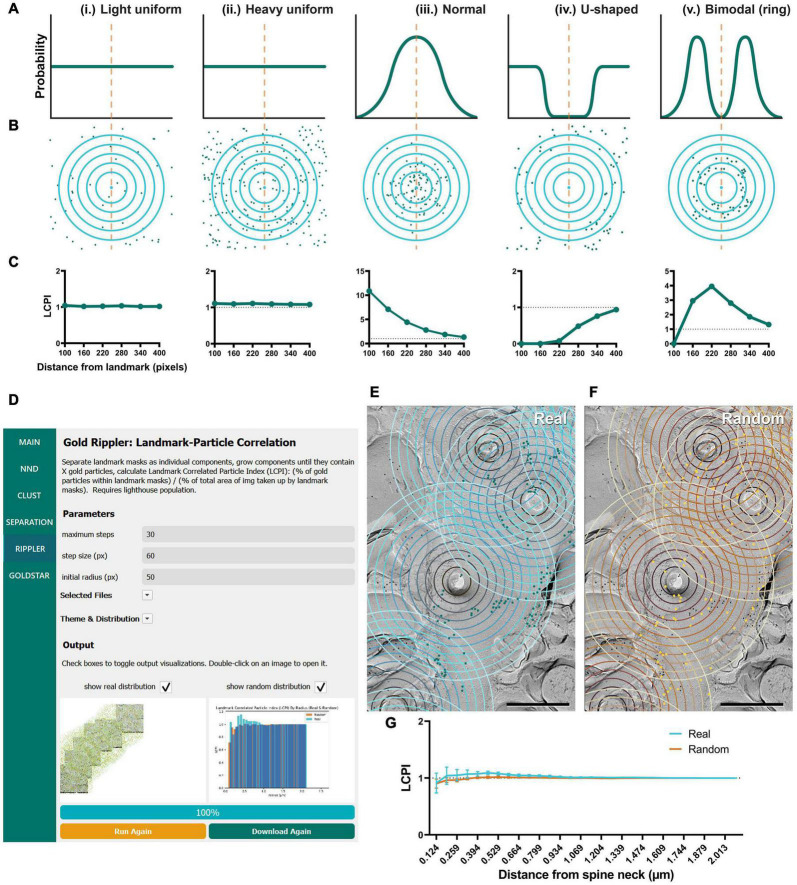
Modeling and application of the Gold Rippler workflow, used for analysis of particle bias toward or away from a landmark feature (e.g., dendritic spines). **(A)** Several theoretical probability distributions that particles may have relative to a landmark feature, and **(B)** a simulation of particle positions using those probability distributions. The orange dashed line indicates the position of the landmark, and blue concentric circles (ripples) radiating outwards indicate binned distances from the landmark. Particles are shown as green dots. (i) Light uniform random and (ii) heavy uniform random distributions of particle positions are examples of zero-bias distributions. (iii) Normal, (iv) U-shaped, and (v) Bimodal distributions of particle positions show some bias relative to the landmark feature. **(C)** Simulated LCPI curves for each distribution (average of 100 iterations). Light and heavy uniform random particle distributions both exhibit a constant LCPI of one regardless of distance from the landmark. A normal distribution of particles around a landmark can be expected to produce an LCPI curve that begins at a high value, then slowly decreases to approach one. A U-shaped distribution can be expected to produce a curve that starts near zero and increases to approach one. A bimodal or ring distribution can be expected to start at or below one, increase past it, then decrease again to approach one. Gray dotted lines indicate an LCPI of one. Random distributions can be expected to match the uniform random distributions shown by (i, ii). **(D)** GIO interface showing the Gold Rippler workflow. Parameters show additional fields for setting the maximum number of steps (ripples), and the step size (distance between ripples) in pixels. A labeled image of the ROI is displayed along with a histogram of the LCPI for real and/or random distributions. **(E)** The Gold Rippler output of a portion of the Purkinje cell dendrite ROI image shown in Figure 5A. Dendritic spine neck cross-fracture is used as the landmark feature, and several blue concentric “ripples” radiate from each. **(F)** The Gold Rippler output for a random distribution of particles (yellow) superimposed on the same ROI. **(G)** The LCPI curve for real and random particle distributions using dendritic spine neck cross-fracture as landmark features. Dots and error bars represent mean ± S.E.M. Dotted line indicates an LCPI of one. *n* = 11 ROIs. Scale bars: 500 nm.

In addition to testing GIO and obtaining the same measurements on Purkinje dendrites, we were interested in developing new methods for examining the relationship of distributions of membrane proteins to structural landmarks (e.g., the cross-fracture of a dendritic spine). Gold Rippler, a unique analysis method included in the package, was developed for this purpose (Figure 6). For each ROI (e.g., a dendritic segment), it produces a metric we term the LCPI, which compares the fraction of particles within a circle of a set radius from the landmark to the relative area of ROI covered by the ripple. The LCPI is measured at increasing binned distances from the landmark, in pixels, corresponding to the rings seen in Figure 1C. A graph is then produced in which the LCPI begins below, at, or above one, depending on the positional bias of particles relative to the landmark, and approaches one as distance from the landmark increases and a greater fraction of particles and ROI area are covered (Figure 6D, lower right). This provides a visual representation of positional bias of particles toward or away from the landmark, or no bias at all. To perform this workflow, the only additional information required is the coordinates of the landmarks, which are loaded into GIO from a separate CSV file (see section “Methods”).

To validate Gold Rippler as a new method for analyzing gold particle distribution, we performed simulations to obtain LCPI curves of a variety of possible particle distributions around a landmark feature, such as a dendritic spine (Figure 6). The simplest expected distributions include (i) light uniform (55 particles), (ii) heavy uniform (220 particles), (iii) normal/Gaussian (110 particles), (iv) U-shaped, and (v) bimodal (ring) distributions; therefore, we simulated gold particles scattered around a single landmark feature using these distributions in 400 × 400 pixel area, and obtained the LCPI graph based on the average of 100 iterations (Figures 6A–C). In cases where particles do not show a biased distribution around the landmark, a uniform random distribution could be expected. The number of particles at the membrane is dependent on the nature of the target proteins and the labeling efficiency of the antibodies used, which is variable; we therefore modeled both sparse and dense distributions of gold particles around the landmark. The results showed that LCPI was resistant to differences in particle density when averaged across 100 iterations, as the curve generated by both is identical [model (i) vs. (ii)]. For a random uniform distribution, the LCPI would be constant as the fraction of gold particles in the ripple equals the fraction of area that the ripple occupies in the whole ROI. Alternatively, when the particle distribution is biased toward a landmark feature, falling off steadily, the distribution of their positions might be normal, with the center of the curve placed at the center of the landmark [model (iii)]. With this distribution, the LCPI begins well above one as the density of particles within the nearest circle is greater than the density outside. As the circle radius increases, the LCPI approaches one (Figure 6Ciii). The counterpart to this distribution is a U-shaped distribution, where particles are excluded from a region near the landmark [model (iv)]. In this case, the LCPI begins well below one near the landmark, then slowly increases to one (Figure 6Civ). Finally, particles may exist in a ring localized at a certain distance from the center of the landmark [model (v)]. In this case, the LCPI starts at or below one, increases past it, then decreases again to approach one (Figure 6Cv).

As proof of concept, we ran Gold Rippler with the replica images labeled for the Ca_*V*_2.1 channel on Purkinje cell dendrites using the center of spines as landmark features. Ca_*V*_2.1 is known to regulate synaptic competition on cerebellar Purkinje cells (Miyazaki et al., 2004; Hashimoto et al., 2011) and visualizing the channel distribution relative to spines could reveal a positional bias. We first observed that the gold particles formed scattered clusters on the membrane (Figure 6E). We then calculated the average LCPI for real and random particles from 11 dendrite segment profiles and observed that the curve for both real and random particle distributions most closely matched the uniform model where the value does not deviate from one across bins (Figure 6G, model i and ii). This suggests that though the particles are clustered with one another, on average they uniformly distribute relative to spines in an unbiased manner.

### Gold Star

**FIGURE 7:**
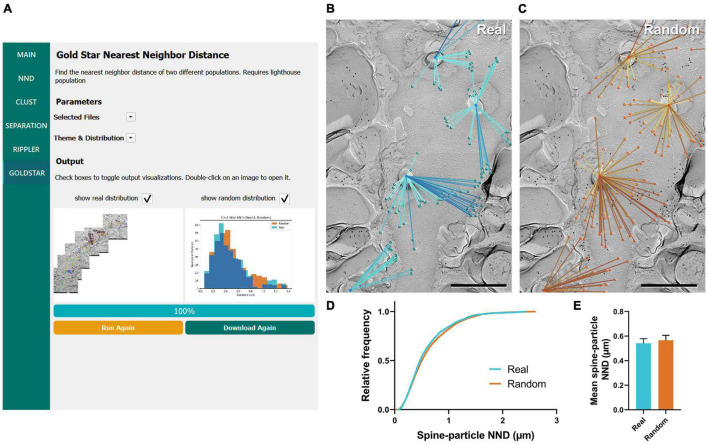
GIO interface of Gold Star as a method for determining NNDs of particles to a landmark feature. **(A)** GIO interface showing the Gold Star workflow. A labeled image of the ROI is displayed along with a histogram of the NNDs to a landmark feature, for real and/or random distributions. **(B)** The Gold Star output of a portion of the Purkinje cell dendrite ROI image shown in Figure 5A. Dendritic spine neck cross-fracture is used as the landmark feature, and lines connect each particle (green) to the center of the nearest spine neck. **(C)** The Gold Star output for a random distribution of particles (orange) superimposed on the same ROI. **(D)** Cumulative frequency distribution of real and random spine-particle NNDs pooled across the 11 ROIs. **(E)** Grand mean spine-particle NND. Error bars represent S.E.M. *n* = 11 ROIs. Scale bars: 500 nm.

The final workflow we developed for gold particle analysis was Gold Star, which was created as a counterpart to Gold Rippler to measure gold particle distances to their nearest landmark feature. This method uses Euclidean distances and therefore is less accurate when dendrites have a high degree of curvature, but unlike Gold Rippler, it produces a concrete metric of particle distances. We ran this workflow to capture the distribution of actual mean distances of Ca_*V*_2.1 channels relative to the dendritic spines of the same Purkinje cell dendrites analyzed in the previous sections (*n* = 11 dendrite segment profiles, Figure 7A). We again randomly placed the same number of particles on each ROI in order to compare real and random distributions to determine whether Ca_*V*_2.1 channel distributions relative to spines deviate from random (Figures 7B,C). We observed no difference in the mean spine-particle NND between real and random (real mean: 0.541 ± 0.04 μm, random mean: 0.567 ± 0.04 μm; *W* = 38, *p* = 0.102, *r* = 0.576, *n* = 11; Figures 7D,E). This provides further evidence that Ca_*v*_2.1 channels do not show a positional bias relative to spines.

### Application of Gold Rippler and Gold Star for Synaptic Analysis

**FIGURE 8:**
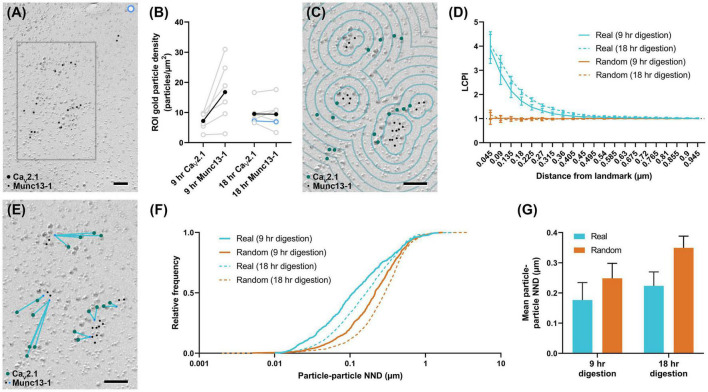
Application of Gold Rippler and Gold Star for synaptic protein analysis. **(A)** Ca_*V*_2.1 and Munc13-1 colocalize at intramembrane protein-rich regions of the calyx of Held membrane which presumably represent the presynaptic active zone. Only a small portion of the larger membrane face (ROI) is shown. **(B)** Overall gold particle density across the calyx of Held membrane for two different SDS-digestion conditions. Gray data points represent individual ROIs and gray lines connect Ca_*V*_2.1 and Munc13-1 densities coming from the same ROI. Solid black dots represent mean densities. Ca_*V*_2.1 and Munc13-1 densities from the ROI partially shown in **(A)** are indicated by light blue circles. **(C)** Zoomed view of the gray box in **(A)** showing a conceptual illustration of Gold Rippler for visual clarity. **(D)** LCPI curve for real and random particle distributions and two SDS-digestion conditions, where Munc13-1 particles are set as landmarks. **(E)** Zoomed view of the gray box in **(A)** showing an illustration of Gold Star. When Munc13-1 is set as the landmark, NNDs (blue lines) are measured between each Ca_*V*_2.1 and the closest Munc13-1 particle (blue dots). **(F)** Cumulative frequency distribution of real and random particle-particle NNDs pooled across the 6 ROIs for each SDS-digestion condition. **(G)** Grand mean particle-particle NND for each SDS-digestion condition. Error bars represent S.E.M. *n* = 6 ROIs for each condition. Scale bars: 100 nm.

To further demonstrate the usefulness of these tools, Gold Rippler and Gold Star were applied for synaptic protein distribution analysis. We examined distributions of Ca_*V*_2.1 and an active zone protein important for synaptic vesicle maturation and fusion, Munc13-1 (Augustin et al., 1999; Karlocai et al., 2021), in wild-type calyx of Held terminals. We compared replicas prepared with two different SDS-digestion conditions, digesting for either 9 or 18 h to obtain the optimal conditions for two proteins having different cellular locations—either membrane integrated or membrane associating (Kaufmann et al., 2021). We analyzed six P-faces of calyx of Held terminals totaling 143.43 μm^2^ for the 9-h digestion condition, and six P-faces totaling 225.88 μm^2^ for the 18-h digestion condition. We found both proteins were localized in intramembrane-protein dense areas, presumably representing the location of the presynaptic active zone (Figure 8A). Replicas digested for 9 h with SDS had slightly lower labeling densities for Ca_*V*_2.1 compared to 18-h digested samples (9-h mean: 7.2 ± 1.2 particles/μm^2^, 18-h mean: 9.6 ± 1.5 particles/μm^2^), while Munc13-1 labeling density was generally higher in the 9-h digested samples (9-h mean: 16.8 ± 4.2 particles/μm^2^, 18-h mean: 9.5 ± 2.0 particles/μm^2^), but these differences were not statistically significant (*t*-tests, Ca_*V*_2.1: *p* = 0.250, *n = 6*; Munc13-1: *p* = 0.156, *n = 6*). Greater variability in the labeling density of Munc13-1 in the 9-h condition was evident (Figure 8B). For the analysis using Gold Rippler, Munc13-1 was set as the landmark feature and particle distribution of Ca_*V*_2.1 relative to it was examined (Figure 8C). We found a strong bias in the distribution of gold particles labeling Ca_*V*_2.1 toward particles for Munc13-1 (Figure 8D), which matched the simulated LCPI curve presented in Figure 6iii. For both digestion conditions, the graph showed gold particles for Ca_*V*_2.1 have an approximately fourfold greater likelihood of localizing within 45 nm of a Munc13-1 particle compared to random, and a twofold greater likelihood within 180 nm. No difference was found in the LCPI of any bins between the two digestion conditions using multiple comparisons tests (all adjusted *p*-values > 0.329), while there was a difference between real and random up to 360 nm away from Munc13-1 particles for the 9-h condition and 675 nm for the 18-h condition. We next applied Gold Star on the same six images from each condition (Figure 8E). The median NND of real Ca_*V*_2.1 relative to Munc13-1, taken from the pooled data, was 111 nm in the 9-h condition and 164 nm for the 18-h condition, while the median for random distributions was 215 and 290 nm, respectively (Figure 8F). When comparing the mean NND of 6 profiles for each condition, we observed no statistical difference in the particle-particle NND between both conditions, though the effect size is considerable (9-h mean: 0.177 ± 0.06 μm, 18-h mean: 0.224 ± 0.05 μm; *U* = 8, *p* = 0.121, *g* = 0.556, *n* = 6). No statistical difference was observed between real and random in the 9-h digestion condition (real mean: 0.177 ± 0.06 μm, random mean: 0.249 ± 0.05 μm; *W* = 19, *p* = 0.063, *r* = 0.905, *n* = 6), but a difference was seen in the 18-h condition (real mean: 0.224 ± 0.05 μm, random mean: 0.350 ± 0.04 μm; *W* = 21, *p* = 0.031, *r* = 1, *n* = 6; Figure 8G). These results indicate Gold Rippler can detect the spatial bias between two proteins despite differences in the labeling efficiency, while absolute distance measurements of the gold particles can be made with Gold Star, though they are more strongly influenced by variation in labeling density.

## Discussion

We developed GIO, an open platform tool for rapid, flexible, and accessible analysis of membrane protein distributions using SDS-FRL. Using the example of Ca_*V*_2.1-labeled calyx of Held terminals, we showed that GIO greatly reduced the time required for image analysis and provided similar values when compared to manual analysis. Further, we demonstrated that GIO could be applied to a variety of biological questions, including the investigation of positional bias of proteins relative to a landmark feature at the subcellular or synaptic level. We discuss the user-friendly nature of GIO, its advantages and limitations, and future development of the package.

### Sodium Dodecyl Sulfate-Digested Freeze Fracture Replica Immunogold Labeling Is a Powerful Technique but Requires Time Consuming Analysis

SDS-FRL provides significant benefits for the two-dimensional visualization of membrane proteins, such as the channels and receptors that control physiological properties of cells. Using this highly sensitive technique, it is possible to study the distribution, quantity, and arrangement of such proteins. However, there are two practical hurdles to overcome for analyzing the immunogold labeled membrane proteins in replica images: (1) How to annotate hundreds or thousands of gold particles, and (2) how to perform numerical analyses on the positions of those gold particles, which represent the distribution of the protein throughout the membrane. The most straightforward approach is to perform the annotation and analysis by hand. In our previous work, we studied the distribution of Ca_*V*_2.1 channels overexpressed on the cell membrane of a giant axon terminal called the calyx of Held labeled by 12 nm immunogold particles (Lübbert et al., 2019). Altogether, we observed six ROIs totaling over 120 μm^2^, and manually annotated 6,122 particles, requiring ca. 20 h of manual labor to annotate and perform our analyses, including NND and clustering. We found simple intensity thresholding using common image editing software was insufficient to speed up analysis, as platinum shadowing of replicas can produce dark regions or spots with the same intensity as gold particles requiring extensive proofreading to remove false positives. Thankfully, tools have been developed in recent years to assist or automatically detect gold particles labeling synaptic proteins, calculate particle NNDs, and compare particle clustering to a random or uniform particle distribution (Szoboszlay et al., 2017; Luján et al., 2018a; Kleindienst et al., 2020). These tools, however, are not suitable to analyze the particularly large stitched images necessary for cellular and subcellular analysis because they were designed primarily to characterize intrasynaptic protein distributions. It is possible to load and analyze large images in Darea, although certain aspects of the interface prohibit subcellular distribution analysis of gold particles. For example, Darea allows manual ROI selection only within a square viewfinder, which prevents analysis of images that do not have an aspect ratio of 1:1. Also, for subcellular image analysis, the ROI is often large and complex, which is not easily demarcated using the built-in polygon selection tool. In addition, due to the large continuous area of the membrane at the calyx of Held terminals, some montaged images to be analyzed can approach 1 GB in size, and cropping the image into manageable pieces is not ideal for efficient analysis. From a morphological perspective, it has been suggested that the distribution of ion channels may be either homogeneous across the entire cell membrane or have a somato-dendritic density gradient, which could be a fundamental factor in controlling the computation of cells or developing their diversity (Nusser, 2009). This can only be directly investigated by studying large and complete cellular images. With this in mind, we recently developed a deep learning-based program for gold particle annotation designed for large stitched images (Jerez et al., 2021), which substantially expedites particle annotation when particle counts are in the thousands. The recent development of machine-learning-based software has also been helpful to annotate gold particles and/or ROIs; however, these do not provide a means for quantification and analysis of particle distributions.

### Gold In-and-Out Was Developed to Contain Several Tunable Workflows for Protein Distribution Analysis

Analyses performed on SDS-FRL images include investigation at both the synaptic (Hagiwara et al., 2005; Tanaka et al., 2005; Masugi-Tokita and Shigemoto, 2007; Kerti et al., 2012; Miki et al., 2017) and cellular-compartment levels (Kulik et al., 2006; Kaufmann et al., 2009; Indriati et al., 2013; Kirizs et al., 2014; Lübbert et al., 2019). The simplest and most commonly used metrics include NND, clustering, and cluster separation, which are used to identify the two-dimensional aggregation of particles (Indriati et al., 2013; Althof et al., 2015; Nakamura et al., 2015; Szoboszlay et al., 2017; Dong et al., 2018; Luján et al., 2018a; Lübbert et al., 2019; Radulovic et al., 2020; Karlocai et al., 2021). We integrated these common analysis methods into GIO in the form of selectable workflows; the user may choose to run their desired workflows at the same time using this feature. Additionally, GIO has several tunable parameters including but not limited to selection of scale, size of clusters, and color scheme, the details of which are stated in the methods. These options allow the user a high degree of customization depending on the research question at hand. The generation of random particle distributions has also been used to compare experimentally labeled particles with random ones drawn in an ROI (Holderith et al., 2012; Indriati et al., 2013; Althof et al., 2015; Szoboszlay et al., 2017). To support this form of analysis, in each workflow we included the ability to generate a random distribution of particles across the ROI. The number of particles used matches the number of real particles by default but can be set to a fixed number when desired.

### Gold In-and-Out Significantly Enhanced Analysis Throughput Compared to a Manual Workflow

We compared our previous manual analysis of overexpressed Ca_*V*_2.1 channels at the calyx of Held, performed using in-house macros for Excel and Fiji, to the automated analysis performed using GIO. We demonstrated that the results were nearly identical, and were consistent regardless of the user; however, they were obtained 2 times faster for NND analysis, and more than 24 times faster for cluster and separation analysis when GIO was applied. In our cluster analysis, we observed a negligible yet consistent and statistically significant decrease in the mean cluster area (∼2% difference) and mean number of gold particles per cluster (∼0.2% difference) using GIO, while particle NND and cluster separation did not differ. Because this minor discrepancy was only seen in cluster measurements, it is likely explained by a small difference in how circles were generated between methods. GIO also provides the extended benefit of comparison to a random distribution within the same workflow. Using this function, we determined that the distribution of overexpressed Ca_*V*_2.1 channel at the calyx of Held was significantly different from random by statistical comparison of particle NNDs and cluster analysis. Cluster separation, however, was not observed to be different from random. Due to the increase in analysis throughput using GIO, we expect the software holds great potential for analyzing changes in cellular protein distribution across developmental stages, under the presence of drug treatments, or following behavioral training. A larger sample size from several experimental conditions can be obtained in less time compared to fully manual analysis.

### Gold In-and-Out Can Be Applied to a Variety of Immuno-EM Analyses

To demonstrate GIO’s flexibility, we analyzed the distribution of immunogold labeled Ca_*V*_2.1 channels on Purkinje cell proximal dendrites in addition to the calyx of Held. Replica images of the calyx of Held exhibit a mostly flat and wide topology, compared to Purkinje dendrite replicas which are often very thin and long, exhibit more extreme curvature, and have a number of dendritic spines along their shaft. Despite this, we were able to successfully analyze the clustering arrangement of Ca_*V*_2.1 channels and showed they were not randomly distributed but instead formed clusters. Interestingly, unlike the calyx of Held, we observed a significant difference in cluster separation from random clustering. It led us to consider whether the channel exhibits a positional bias relative to dendritic spines, therefore we developed a new workflow to examine the biological function-related bias (see next paragraph). As we have shown, GIO lends well to analysis of large images, but it is also suitable to be applied to analysis of synaptic protein distributions, similar to existing software such as GoldExt (Szoboszlay et al., 2017), GPDQ (Luján et al., 2018a), and Darea (Kleindienst et al., 2020). Future applications of GIO include its potential use in analyzing immuno-labeled thin section images, where protein localization can be analyzed in single or serial sections. Furthermore, usage of GIO could be expanded to investigate the distribution or positional bias of organelles in cells from different biological conditions. Any new workflows can be added into GIO as custom modules by adapting existing workflows to fit to the user’s research purpose.

### Gold In-and-Out Contains New Analysis Tools to Study Positional Bias of Protein Distribution

We introduced two new complementary workflows: Gold Rippler and Gold Star. Both workflows are useful for investigating particle distribution bias toward or away from a landmark feature in both subcellular and synaptic analysis. For studying subcellular protein distributions, the landmark could be any morphological feature such as somata, branching points of dendrites, or dendritic spines. For analyzing synaptic protein distributions in either pre- or postsynaptic regions, the relationship between two proteins can be examined. Gold Rippler generates binned areas of a fixed distance interval around the landmark, which we term ripples, to produce a metric of positional bias we term LCPI (see Section “Methods” for details). The experimentally measured LCPI graph generated by Gold Rippler can be compared to several theoretical distributions, allowing for classification of the type of distribution formed around a landmark feature. This metric has some benefits over standard density measurements calculated by the number of particles per unit area—because LCPI is normalized to the number of particles in the ROI and the size of the ROI, data can be pooled across images from different batches of samples. For example, there are often differences in labeling efficiency between replicas, which could be generated by slight differences in SDS-digestion of the tissue. As we showed in Figure 8, LCPI is resistant to these sources of variability because the area covered by each ripple generally scales with the number of landmark features present. Furthermore, if two proteins are associated, they should roughly maintain the same pattern of close proximity regardless of labeling efficiency or particle density, which could be strongly affected by experimental condition. Though it is useful for getting a sense of bias, the LCPI does not provide a concrete measurement of inter-object distances. It also requires averaging across many ROIs ( > 10 large ROIs) to get a meaningful result, since LCPI can be highly variable in the bins nearest to the landmark as those bins have the smallest combined area, and the fraction of particles captured is more variable in smaller areas. Gold Star is a simple NND workflow that measures Euclidean distances between particles and their nearest landmark feature and is useful for investigating the distribution of protein positions. It is, however, less resistant to the characteristically sinuous and curving nature of dendritic profiles and occasionally draws lines that pass outside of the border of the ROI as a result. This is much less of a concern for synaptic analysis, where distances between proteins are shorter.

In the present study, we found that Ca_*V*_2.1 on Purkinje dendrites showed non-random clustering (Figure 6), and the separation between clusters was significantly different from random, implying the distribution may have positional bias relative to a specific feature. Previous research suggested Ca_*V*_2.1 was a key factor in the developmental regulation of climbing fiber-Purkinje cell dendrite synaptic competition in the cerebellum (Miyazaki et al., 2004, 2012). Therefore, we hypothesized the channels may also be preferentially oriented toward dendritic spines, assuming Ca_*V*_2.1-mediated synaptic competition also drives spine formation and/or pruning. We found no evidence of positional bias of Ca_*V*_2.1 relative to spines. It is possible that Ca_*V*_2.1 in the dendritic spine head mediates synaptic competition, rather than channels along the dendrite; alternatively, the molecular mechanisms governing competition occur globally at the dendrite and do not require specific positioning of Ca_*V*_2.1.

To validate our simulation data and show the usefulness of Gold Rippler and Gold Star for analyzing the distribution of two synaptic proteins, we investigated the spatial association between Ca_*V*_2.1 and Munc13-1 at the calyx of Held. Our results not only show a clear positional bias of Ca_*V*_2.1 toward Munc13-1 on the membrane, they support the claim that Gold Rippler is resistant to differences in labeling density caused by differences in labeling efficiency between sample preparation conditions. Measurement of real and random particle-particle NNDs using Gold Star shows a slightly significant difference in the distribution of the 18-h digestion condition but not the 9-h condition, despite the clear trend seen with Gold Rippler’s LCPI curve. We view this as evidence that NND measurements obtained by Gold Star are more susceptible to variations in labeling density.

Another proposed use of Gold Rippler is to set the landmark as the centroid of a synapse (demarcated based on clustered intramembrane proteins), making it possible to detect positional bias toward the edge or center. This is similar in concept to a method developed for the analysis of intrasynaptic distributions of glutamate receptors, which also uses concentric binned areas projected inwards from the edge of the synapse to form a “heat map” of receptor positions (Budisantoso et al., 2013; Rubio et al., 2017). Rather than extending ripples internally, Gold Rippler expands ripples outward from the center. From there, one can compare the distribution of proteins to a randomly distributed population within the synapse.

### Considerations for Subcellular Analysis of Replica Images Using Gold In-and-Out

It is an advantage of SDS-FRL to visualize membranes two-dimensionally, however, performing analyses at a subcellular scale can prove challenging. Because physical fracturing of frozen tissues occurs randomly, it is impossible to entirely capture the complete three-dimensionality of biological features, which means replicas have hidden features (e.g., dendritic spines) that are missed by potential analyses. The inability to account for hidden landmarks may introduce a source of bias in the distribution of gold particles measured using subcellular analysis. Furthermore, as the area of membrane to be analyzed increases, the likelihood of encountering interruptions such as cross fracture or patches of opposing membrane leaflet (i.e., P-face or E-face) which conceal parts of the membrane increases. The effect of this phenomenon is lessened by having a sufficiently large sample size—of both membrane area and number of cells sampled—in which case any error relating to the boundary of the ROI should become minimized.

### Accessibility and Current Limitations of Gold In-and-Out

We designed GIO with one primary objective: to create a package that is comprehensive, user friendly, easy to access, and customizable. We found that installation of existing programs requires a certain level of coding knowledge, specific versions of Matlab or Python, or familiarity with open-source software to run, which can prevent use by researchers who are not knowledgeable on the subject. Almost no training is required for first-time users of GIO due to its user-friendly GUI, and no outside software is needed as it is fully self-contained, requiring no installation process. Moreover, GIO requires no coding experience to run and collect data. It opens in seconds and can provide data output in as little as 1–5 min depending on the number of particles in the image and the workflows selected. Importantly, GIO precludes any personal bias by automating analyses and as a result, the analyses performed are consistent regardless of the user. Despite its usefulness, GIO has room to grow. Currently, one image is analyzed at a time, which requires the user to compile all result outputs in another program and use additional software for statistical analysis. This is not a major limitation when analyzing a dozen or so images, but is inhibitive when analyzing several dozen or more. In future iterations of the program, we aim to integrate multiple image upload capabilities to make analysis faster and more convenient for the user. Due to the extensive breadth of statistical analysis possible and difficulty of such an undertaking, we do not currently have plans to integrate statistical analysis directly into GIO, and data must therefore be analyzed in a separate dedicated software package. However, if the user desires to add or modify existing modules, we aimed to create an easy-to-read, organized, and open repository for the source code, which is provided on Github. GIO can be downloaded from Github at https://github.com/mpfi-dsp/GoldInAndOut.

## Data Availability Statement

The original contributions presented in the study are included in the article/supplementary material, further inquiries can be directed to the corresponding author/s.

## Ethics Statement

The animal study was reviewed and approved by the Max Planck Florida Institute for Neuroscience Animal Care and Use Committee.

## Author Contributions

NK conceived and supervised the project. NK, DG-G, DJ, and SG designed the program. DJ created code for the initial NND and Gold Rippler implementation. SG wrote other code and formatted the GIO software. DG-G and CT tested GIO. DG-G, CT, and SA ran GIO and collected the data. CT performed statistical analysis and created figures. NK and CT wrote the manuscript. All authors contributed to the article and approved the submitted version.

## Conflict of Interest

The authors declare that the research was conducted in the absence of any commercial or financial relationships that could be construed as a potential conflict of interest.

## Publisher’s Note

All claims expressed in this article are solely those of the authors and do not necessarily represent those of their affiliated organizations, or those of the publisher, the editors and the reviewers. Any product that may be evaluated in this article, or claim that may be made by its manufacturer, is not guaranteed or endorsed by the publisher.
